# The key role of cheaters in the persistence of cooperation

**DOI:** 10.1186/s12915-025-02492-5

**Published:** 2026-01-03

**Authors:** Sanasar G. Babajanyan, Yuri I. Wolf, Eugene V. Koonin, Nash D. Rochman

**Affiliations:** 1 Computational Biology Branch, Division of Intramural Research, National Library of Medicine, National Institutes of Health, Bethesda, 20894 MD USA; 2 Institute for Implementation Science in Population Health, City University of New York, New York, 10027 NY USA; 3 Department of Epidemiology and Biostatistics, Graduate School of Public Health and Health Policy, City University of New York, New York, 10027 NY USA

**Keywords:** Evolution of cooperation, Multilevel selection, Social traits, Altruism

## Abstract

**Background:**

Evolution of cooperation is a major, extensively studied problem in evolutionary biology. Cooperation is beneficial for a population as a whole but costly for the bearers of social traits such that cheaters enjoy a selective advantage over cooperators. Here, we focus on coevolution of cooperators and cheaters in a multi-level selection framework, by modeling competition among groups composed of cooperators and cheaters. Cheaters enjoy a reproductive advantage over cooperators at the individual level, independent of the presence of cooperators in the group. Cooperators carry a social trait that provides a fitness advantage to the respective groups.

**Results:**

In the case of absolute fitness advantage, where the survival probability of a group is independent of the composition of other groups, the survival of cooperators does not correlate with the presence of cheaters. By contrast, in the case of relative fitness advantage, where the survival probability of a group depends on the composition of all groups, the survival of cooperators positively correlates with the presence of cheaters. Increasing the strength of the social trait alone fails to ensure survival of cooperators, and the increase of the reproduction advantage of the cheaters is necessary to avoid population extinction. This unexpected effect comes from multilevel selection whereby cheaters at the individual level become altruists at the group level, enabling overall growth of the population that is essential for the persistence of cooperators. We validate these theoretical results with an agent-based model of a bacterial biofilm where emergence of the cooperative trait is facilitated by the presence of cheaters, leading to evolution of new spatial organization.

**Conclusions:**

Our results suggest that, counterintuitively, cheaters often promote, not destabilize, evolution of cooperation.

**Supplementary Information:**

The online version contains supplementary material available at 10.1186/s12915-025-02492-5.

## Background

Cooperation is a ubiquitous social trait, observed at every level of biological organization, spanning viruses [1, 2], bacteria [3–6], and animals [7, 8], and is essential for the emergence and survival of complex organisms and communities including human societies [9–11]. Understanding the underlying mechanisms of major transitions in evolution [12–14], such as emergence of the first cells [15–17] and multicellular organisms [18–20], requires elucidating the nature of the selective pressures that bring about cooperative (social) traits and support their persistence.

Cooperation requires individual agents to act in the interest of the community, which is not necessarily aligned with the interest of those agents themselves. Consequently, cooperative systems are vulnerable to cheaters, agents which do not contribute to but still benefit from the cooperative behavior of other group members. The emergence of cheaters imposes a relative fitness disadvantage on remaining cooperators which can eventually lead to complete loss of cooperative traits throughout the population [21–25].

Many mechanisms supporting the emergence and persistence of cooperation, which are robust against cheating, have been theoretically described and some have been empirically characterized, including but not limited to kin selection [26–29], reciprocal interactions [28, 30–33], non-homogeneous environmental factors [34–38], indirect reciprocity [28, 39–41], and structured interaction, that is, heterogeneous interactions at the individual level [42–47], and homogeneous interaction between individuals within groups in a group-structured population [48–52].

In the multilevel selection framework, which models interactions between individual agents as well as interactions between groups, potentially at multiple hierarchical levels, conflict between individual and group level selection can appear whereby a trait is disadvantageous on the individual level but advantageous on the group level, or vice versa [15, 51, 53–55]. Addressing this conflict, the emergence and persistence of social traits, that are disadvantageous at the individual level, can be enabled by restricting interactions with cheaters or by other mechanisms resulting in fitness advantage of cooperation at the group level [15–17, 48–52].

As in the case of single-level selection, in multilevel selection scenarios, the presence of cheaters is typically associated with a negative impact on the fitness of cooperators and most prior work has focused on exploring mechanisms that promote resistance to and elimination of cheaters as the only path to the survival of cooperators. Here, we demonstrate the counter-intuitive phenomenon whereby, in the context of multilevel selection, the emergence of cheaters can promote, and can even be essential, for the long-term survival of cooperators.

These dynamics can emerge as the result of partitioning any population into two compartments: the bulk and the interface. The individuals in the bulk are protected by those at the interface from environmental stressors leading to elevated mortality rates. These stressors could include pathogen exposures, predation, or physical factors like shear stress. The fraction of the population at this interface may be inversely dependent on the total size of the population. For example, consider the growth of a rainforest which strongly influences its own weather patterns [56]. At the edges of the rainforest, individual plants are more susceptible to drought than those toward the center and as the forest grows, the fraction of the plants on the edge decreases.

Now let us assume that such a compartmentalized population is additionally subdivided into two types of individuals: social individuals and asocial individuals (which may often be characterized as cooperators and cheaters, respectively). The social individuals modify their local environment to reduce environmental stress through the costly production of a public good. In particular, consider a public good which binds individuals closely together, maintaining the integrity or even reducing the size of the interface. For example, bacteria in biofilms secrete extracellular matrix proteins which tie them to a substrate [57], reducing cell loss due to physical agitation or exposure to predators, antibiotics, or viruses. The synthesis of these proteins is energetically costly. Similarly, tumor cells display a reversible phenotypic switch, the Epithelial–Mesenchymal transition (EMT) [58], where interface integrity is better maintained in the epithelial state at the cost of reduced proliferation and migration. Alternatively, consider the evolution of sociality in prey mammals [59]. Sociality gives rise to foraging strategies that support protected groups with a clearly defined interface at the cost of a prolonged period of a fully-dependent juvenile state.

In each of these diverse systems, we can immediately consider the following: when alone, asocial individuals are able to survive in harsher environments than social individuals because they do not pay the cost of the social trait. Indeed, asocial bacteria do not synthesize matrix proteins; tumor cells in the Mesenchymal state are more likely to migrate; mammals that do not rear their young are able to more quickly scale reproduction. Here, we explore how, under the right conditions, the presence of asocial individuals can protect pioneering social individuals and at the same time, social individuals are able to modify their environment so that, as the population grows and the interface relatively shrinks, sociality is an evolutionary stable strategy robust to domination by asocial “cheaters”.

Here, we present two models. First, we fully characterize a theoretical model that yields analytical solutions. This model is even more general than the situation described above, but is subject to a strong mathematical constraint, namely that the mortality rate (over groups, as defined in the following section) is not just inversely proportional to the total population size but constant over time (and consequently, is governed by a power-law dependence on total population size). Second, we present a simplified agent-based simulation of the first of the examples described above, the biofilm, explicitly demonstrating that we can recover the key results when this mathematical constraint is relaxed.

## Model

Our model relies on three principal assumptions: the utility of a multilevel selection modeling framework; selection at the individual level is frequency independent; and selection at the group level is frequency dependent. In our view, the validity and biological relevance of modeling multilevel-selection is evident in the success of prior work explaining and predicting the emergence of specific, diverse cooperative behavior including the phenomena described in the introduction. Consequently, we expect conditions suitable for the application of such a framework to be pervasive across most if not all biological systems.

Within the model, individuals are either cooperative or noncooperative. Cooperative individuals carry a growth-costly trait. This cost is always paid, independent of the absolute number or fraction of cooperators and asocial individuals in the local group, or the whole environment. This framework is broadly representative of any biological system in which a subpopulation of cooperators invest energy in the production of public goods; however, in general this cost may be variable, (see Discussion). Below, we highlight the production of extracellular matrix proteins by cooperators in bacterial biofilms as one example (see Fig. 6).

In contrast, within our model, groups are relatively, but not absolutely, more likely to survive when they contain a relatively larger fraction of cooperators compared to other groups. This behavior arises from the implementation of a constant rate of group death, independent of the total number of groups. This idealized case in which the probability of group death is inversely proportional to the total number of groups is likely not observed in many real biological systems. However, it represents only the limiting case of a much more general condition, where the fraction of groups susceptible to death grows slower than the total number of groups and, consequently, the probability of group death declines with the total number of groups. We expect this more general condition to be widely observed in many real biological systems where predation or other forms of extrinsic mortality primarily operate at the surface of spatially structured populations. We provide one such particular example of biofilm growth (see Fig. 6).

The adoption of these strong simplifying assumptions are what enable us to support a generalizable, theoretical framework to evaluate the evolution of cooperative behavior that is not specific to any one physical system. Introducing greater model complexity, for example with the inclusion of frequency-dependent selection at the individual level, would likely prohibit the identification of the analytical results we report which we believe are useful tools to build intuition and support more specific models reflecting individual biological systems.

We consider competition between groups composed of social (*A*) and asocial (*B*) individuals that differ in their reproduction rates such that the social trait is associated with a growth cost. In exchange, the survival probability of a group increases with the fraction of cooperators (social individuals) within that group. We assume that the reproduction advantage of asocial individuals (*B*) is independent of the composition of a group. That is, we assume frequency-independent reproduction at the individual-level: social individuals provide a fitness advantage on the group level, while all per capita reproduction rates are independent of the composition of groups. The last assumption differs from the setup most commonly used in evolutionary game theory, where the fitness of both social and asocial individuals is frequency-dependent at the individual level (in the simplest case, defined by a matrix game such as the prisoner’s dilemma) as is commonly considered in the context of evolution of cooperation [8, 28, 31, 60].

**Fig. 1:**
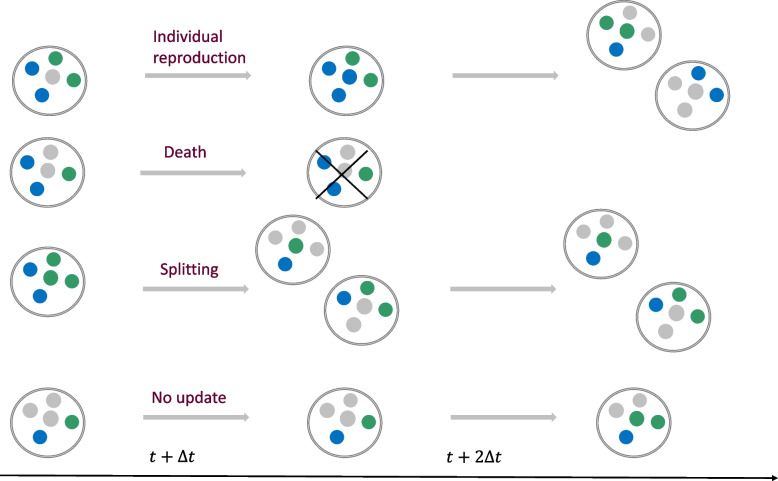
Schematic representation of the elementary processes occurring in the model in each time step $\Delta t$. Each group may consist of social (*A*) and asocial (*B*) individuals and available resources, blue, green and gray balls, respectively. The total number of individuals and resources in each group, *K*, is fixed. Individuals within each group reproduce by consuming available resources in the group. The reproduction probabilities of social and asocial individuals in a given group is given by (1) and (2), respectively. Group death eliminates both individuals and resources within the group. Social individuals provide a fitness advantage to the group, relative or absolute, by decreasing the death probability of the group. The death of a group occurs at time *t* with probability (7) and (12) for relative and absolute fitness advantage cases, respectively. Group splitting occurs whenever any group contains *K* individuals (blue and green balls), and no resources (gray balls). Splitting is binary, and results in the random allocation of all individuals in the parent group into the daughter groups. The probability that a group splits at time *t* is given by (4)

Here, the changes in group number and composition are modeled in parallel over discrete time steps during which individual reproduction in each group, group splitting, and group death may all occur, including the possibility that no event occurs, see Fig. 1. We emphasize that group splitting refers to group proliferation (through subdivision and colonization at the individual level) in the abstract and does not invoke any specific physical process. Below, we introduce the processes occurring in each discrete, fixed-interval time step, $\Delta t=1$, in detail.

###  Individual level interactions

Each group has a fixed number of sites, *K*, which can be occupied by resources, *R*, an *A* individual or a *B* individual. Individuals compete for available resources within each group. Let us denote the number of *A* and *B* individuals within a group *j* by $n_{j,A}$ and $n_{j,B}$, respectively. The resources available in this group are $K-n_{j,A}-n_{j,B}$. We assume that the resources within each group are defined at the time of the formation of each group, and no resource intake takes place until group splitting. Group splitting, through division, occurs when all resources are exhausted in the group $n_{j,A}+n_{j,B}=K$, whereas individual reproduction can occur only if there are available resources in the group, $n_{j,A}+n_{j,B}<K$.

The probability of an individual reproduction event is proportional to the amount of available resources in the group $1-\frac{n_{j,A}+n_{j,B}}{K}$, which decreases as the number of individuals in the group approaches the splitting threshold *K*. We assume that the reproduction of social and asocial individuals is proportional to their fractions in the group, yielding the following transition probabilities

1 $$\begin{aligned} & A+R\rightarrow 2A,\quad T(n_{j,A}, n_{j,B}\rightarrow n_{j,A}+1, n_{j,B})=\frac{n_{j,A}}{K}\left(1-\frac{n_{j,A}+n_{j,B}}{K}\right),\end{aligned}$$

2 $$\begin{aligned} & B+R\rightarrow 2B, \quad T(n_{j,A}, n_{j,B}\rightarrow n_{j,A}, n_{j,B}+1)= b \frac{n_{j,B}}{K}\left(1-\frac{n_{j,A}+n_{j,B}}{K}\right). \end{aligned}$$

In (2), $b>1$ specifies a relative reproductive advantage of asocial (*B*) over social (*A*) individuals and so we only consider the range $b\ge 1$. The transition probabilities (1) and (2) sum to at most 1 and with probability $1- T(n_{j,A}, n_{j,B}\rightarrow n_{j,A}+1, n_{j,B})-T(n_{j,A}, n_{j,B}\rightarrow n_{j,A}, n_{j,B}+1)$ no reproduction occurs within the group at the given time step. The sum of transition probabilities is maximized when $n_A=0$ and $n_B=\frac{K}{2}$ and so it follows $b\le 4$. Note, the composition of all groups are updated in parallel every time step.

###  Group level interactions

We will denote the compositions of all groups in the population, by $N_g(t)$. A group *j* alive at time $t-\Delta t$ would die at time *t* with probability:

3 $$\begin{aligned} P_{j,\textrm{death}}(t)=\mu g_j(\boldsymbol{G}(t)) \end{aligned}$$

where $\mu$ is the probability that any group death occurs in a given time step. The effect of the social trait is incorporated through $g_j(n_{1,A}(t),n_{1,B}(t),...,n_{j,A}(t),n_{j,B}(t),...n_{N_g,A}(t),n_{N_g,B}(t) \equiv g_j(\boldsymbol{G}(t))$, which is a function of $N_g(t)$, in general. Thus, group elimination works as follows: first, one decides whether any group elimination may occur at the given time step by probability $\mu$, then the functions $g_j(\boldsymbol{G}(t))$ define which group will be eliminated if any. $g_j(\boldsymbol{G}(t))$ specifies the relative fitness advantage of group *j*. In the neutral case, that is in the absence of a social trait, $g_j(\boldsymbol{G}(t)) \equiv g(\boldsymbol{G}(t)) =\frac{1}{N_g(t)}$ and $\sum ^{N_g(t)}_{j=1}{g_j(\boldsymbol{G}(t))}=1$. The latter condition ensures that one of the groups dies with probability $\mu$ at every time step. We will relax this assumption in the context of absolute fitness advantage where the probability that any group may die may be less than $\mu$, $\sum ^{N_g(t)}_{j=1}{g_j(\boldsymbol{G}(t))}\le 1$.

Each time step, group splitting occurs with probability 1 whenever at least 1 group has reached the splitting threshold, *K*, and the total number of groups, $N_g(t)$, remains below the environmental carrying capacity, $K_g$. If $N_g(t) < K_g$, one among the groups that have reached the splitting threshold *K* is randomly chosen to reproduce. The probability that group *j* splits (a birth event) at time *t* is given by:

4 $$\begin{aligned} P_{j,\textrm{birth}}(t)=\frac{D_{n_j(t),K}}{\sum _{k=1}^{N_{g}(t)} D_{n_k(t),K}}\Theta (K_g-N_g(t)) \end{aligned}$$

where $D_{k,l}=1$ if $k=l$ and 0 otherwise, denotes the Kronecker delta function to avoid confusion with the definition of $\delta (t)$ used throughout, and $\Theta (x)=1$ if $x>0$ and 0 otherwise. Note that $K_g$ impacts group reproduction, but not death probabilities. If no group has reached *K*, $P_{j,\textrm{birth}}(t)=0$. Note that, unlike group death, the probability of group splitting is independent of group composition and unaffected by the social trait.

Splitting of a parent group with composition $(n_{j,A},n_{j,B})$, such that $n_{j}\equiv n_{j,A}+n_{j,B}=K$, results in the formation of two daughter groups

with compositions $(m_{j,A},m_{j,B})$ and $(n_{j,A}-m_{j,A},n_{j,B}-m_{j,B})$, respectively, where $m_{j,A} = U(0,n_{j,A})$ and $m_{j,B} = U(0,n_{j,B})$ are sampled from uniform distributions. The uniform distribution is used here for the sake of simplicity. While a Poisson distribution would be more natural, it is highly unlikely this choice would qualitatively determine the behavior of the system and would add substantial complexity to the analytical solutions for the model. If $m_{j,A}=m_{j,B}=0$ the corresponding group is immediately eliminated resulting in an abortive splitting event with probability $\sim \frac{1}{n_{j,A} n_{j,B}}$.

The assumption that the death probability is time and group-number independent reflects the limiting case of a much broader family of systems for which the growth rate of the total population exceeds the growth rate of the subpopulation susceptible to death. The agent-based biofilm proliferation process presented below illustrates one such example.

### Survival and growth of groups in the absence of the social trait

To characterize the behavior of the model under conditions of competition between social and asocial individuals, we must first establish the conditions under which groups proliferate in the absence of the social trait, that is $g_j(\boldsymbol{G}(t)) =\frac{1}{N_g(t)}$ in (3). We may recall within each time step, at most one group death occurs with probability $\mu$ (both in the relative fitness case generally and in the absence of the social trait). In the limiting case where the population is homogeneous with the frequency-independent reproduction scale factor $b\ge 1$. Then, $b=1$ and $b>1$ corresponds to homogeneous populations of social and asocial individuals, respectively (although we assume no social trait here, the provided analysis holds in the presence of social trait too due to the frequency-independence assumption of the individual level trait).

Our goal is to predict the outcome of population dynamics, that is extinction or proliferation, based on the initial number of groups $N_g$ and remaining model parameters. The survival probability of a group at time *t* is $\delta (t) = 1 - P_{\textrm{death}}(t)=1-\frac{\mu }{N_g(t)}$, which monotonically decreases with $N_g(t)$. If at time $t>>0$, $\delta (0) < \delta (t)$, then $N_g(t)>N_g(0)$, thus the population of groups has grown. Conversely, if $\delta (0)> \delta (t)$, then the population has declined. Substituting $N_{g}(0)\equiv N_g$ for $N_g(t)$ yields a bound for group survival probability (upper under conditions of decline, and lower under conditions of growth): $\delta \equiv 1-P_{\textrm{death}}(0)= 1- \frac{\mu }{N_g}$ which supports the construction of several analytical approximations that agree with simulation.

The growth of the population of groups requires that, on average, more than one daughter group survives to split again. As each group splits into two daughter groups, this requires that the probability of reaching the splitting threshold is greater than the probability of death.

We denote the probability of reaching the splitting threshold from an arbitrary initial state $0<n<K$, $\psi _n(\delta ,b,K)$. Using the time-independent bound on $\delta$ described above, a closed form expression may be obtained by recursion (see (16) in the Methods “Proliferation in neutral case” section). Averaging over all possible initial states $\langle \psi (\delta ,b,K)\rangle =\frac{1}{K-1}\sum _n^{K-1}\psi _n$ (which also reflects the random allocation of all individuals of the parent group into the daughter groups) yields the following condition. The population of groups will proliferate if $\langle \psi (\delta ,b,K)\rangle>1/2$. At equality $\langle \psi (\delta , b, K)\rangle =1/2$, that is reproduction and death are equally likely on average, in the limit of high survival probability, $\delta \sim 1$, from $\psi _n(\delta ,b,K)$ we obtain the following relation between the model parameters (see the Methods “Proliferation in neutral case” section and Additional File 1: SI.B)

5 $$\begin{aligned} \frac{\mu }{N_g}=\frac{K-1}{2 H_{K-1} K^2} b, \end{aligned}$$

where $H_{n}=\sum _{k=1}^n \frac{1}{k}$ is the *n*th harmonic number. The limit of high survival probability $\delta = 1-\frac{\mu }{N_g}\sim 1$ implies $\mu /N_g \sim 0$.

**Fig. 2:**
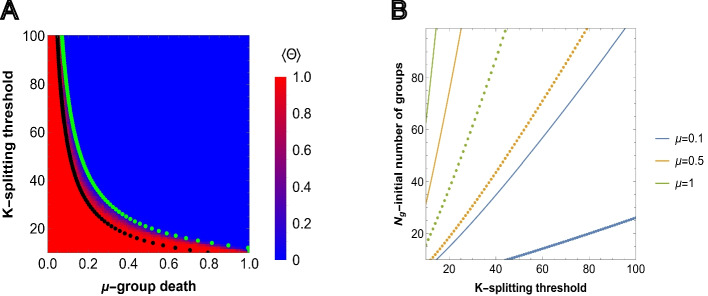
Survival of groups depending on the model parameter values. **A** predictions obtained from (18) and simulation for $b=1$ and fixed initial group size $N_{g}=50$. Each cell shows the value of (6) obtained in simulations, $M=50$ independent realizations, where the sampling is done at $T=1000$. Steps for each pixel in the heatmap are chosen with $\Delta \mu =0.01$ and $\Delta K=1$. The black and green dotted lines show the values of $\mu$, for fixed *K* and $N_g$, corresponding to $\langle \psi \rangle =\frac{1}{2}$ obtained under the assumption of high survival probabilities (5), and without that assumption, respectively. **B** relation between the initial number of groups $N_g$ and splitting threshold of a group *K* for different values of group death probability $\mu$ and splitting threshold $K\in [10,100]$ obtained from (5), below which the population goes extinct. Solid and dotted curves show the threshold values for $b=1$ and $b=4$ reproduction scale factors, respectively

From (5), it follows that for a given initial number of groups $N_g$, reproduction scale factor *b*, and splitting size threshold *K*, there exists a corresponding threshold value of $\mu$ above which the population is more likely to go extinct than to reach environmental carrying capacity $K_g$ because group reproduction is less likely than group death. Similarly, (5) provides a threshold relation between the number of groups $N_g$ and splitting threshold *K* for a given group death probability $\mu$ and reproduction scale factor *b*. That is, we may identify the minimum number of initial groups $N_g$ that admits the proliferation of the population subject to the splitting threshold *K* for fixed $\mu$ and *b*. These two relations are presented in Fig. 2A (black dotted lines) and in Fig. 2B, respectively.

The relation (5) provides the lower bounds for the model parameters, obtained in the limit of high survival probabilities $\delta \sim 1$ that admit group survival and proliferation (see Additional File 1).

Complementary to (5), we consider $\langle \psi (\delta ,b,K)\rangle =\frac{1}{2}$ without imposing the $\delta \sim 1$ condition. The green dotted line in Fig. 2A shows the pairs of $(\mu , K)$, where $\mu$ is found by solving $\langle \psi (\delta ,b,K)\rangle =\frac{1}{2}$ for each integer value of $K\in [10,100]$, for $N_g=50$.

We compared the predictions obtained from $\langle \psi (\delta ,b,K)\rangle>1/2$ with individual based simulations. For the simulations, we used an indicator function, that shows whether any group is present in the environment after time *T* or not, $\Theta (N_g(T))=1$ if $N_g(T)> 0$, and $\Theta (N_g(T))=0$ otherwise.

6 $$\begin{aligned} & \langle \Theta \rangle = \frac{1}{M}\sum _{\alpha =1}^M \Theta _{\alpha }(N_g(T)) \end{aligned}$$

Thus, $\langle \Theta \rangle =1$ means that extinction was never observed, up to time *T*, in each run of the simulation. The comparison of the simulation results and prediction of (18) is presented in Fig. 2A. In Fig. SI1, results are shown for $N_g=25$ and $N_g=100$.

As expected, lower splitting threshold *K* and death probability $\mu$, along with larger initial number of groups $N_g$, increases the probability of proliferation. Conversely, the higher the splitting threshold of groups (that is, the larger the individual groups), the larger the number of initial groups necessary for the population to survive and proliferate. Increasing the reproduction advantage *b* in (5) expands the region where group proliferation is possible.

The curves obtained via $\langle \psi (\delta ,b, K)\rangle =\frac{1}{2}$ and (5) together accurately describe the results of agent-based simulation, providing upper and lower bound estimates for the model parameters that admit group proliferation.

## Results

### Relative fitness advantage of groups with the social trait

Here we model the social trait to provide a relative advantage to groups with a greater fraction of *A* individuals by decreasing the probability of group death:

7 $$\begin{aligned} P_{j,death}(t)=\mu \frac{1-a\frac{n_{j,A}(t)}{n_{j,A}(t)+n_{j,B}(t)}}{\sum _{l=1}^{N_g(t)} 1-a\frac{n_{l,A}(t)}{n_{l,A}(t)+n_{l,B}(t)}}, \end{aligned}$$

where *a* is the strength of the social trait. From (7), it follows that the survival probability of a given group depends on both the total number and the composition of all groups, thus representing density and frequency dependent selection on the group level. The social trait function was chosen to recover a well-known fitness-dependent birth-death process ([48, 60–62]), for the case when the group elimination probability is small, $\mu \ll 1$, the population reaches environmental carrying capacity $K_g$, and the model parameters are in the region admitting proliferation of both all-*A* ($b=1$) and all-*B* ($b=4$) groups (see Fig. 2B, further details are provided in the Methods “Relative and absolute fitness cases for µ ≪ 1” section). We first consider the limit where all groups are exclusively composed of one type of individual, *A* or *B*, and then provide the results for the case of initially heterogeneous groups.

Relaxing the $\mu \ll 1$ condition, in any population of homogeneous groups, where the total number of groups $N_g$ can be below the environmental carrying capacity, the survival probabilities of all-*A* and all-*B* groups are equal to:

8 $$\begin{aligned} \delta _A= 1- \frac{\mu (1-a)}{N_g- a N_{g,A}},\quad \delta _B=1- \frac{\mu }{N_g- a N_{g,A}}, \end{aligned}$$

From (8) it follows that for a fixed number of groups, $N_g$, the probability of any group to be eliminated from the population increases with the fraction of all-*A* groups in the population $\frac{\partial \delta _i}{\partial N_{g,A}}<0, ~i=A,B$.

Indeed, for any of the all-*B* groups, the presence of all-*A* groups increases the likelihood of death due to the survival advantage of all-*A* groups (7). Similarly, the presence of any other all-*A* group in the population decreases the relative advantage of each all-*A* group. The survival probabilities also depend on the social trait strength, *a*, and increasing *a* has the opposite effect on the survival probabilities of all-*A* and all-*B* groups, that is, $\frac{\partial \delta _A}{\partial a }>0$ but $\frac{\partial \delta _B}{\partial a}<0$.

The average probabilities of reaching the splitting threshold, *K*, for homogeneous groups $\langle \psi _A(\delta _A, b=1, K)\rangle$ and $\langle \psi _B(\delta _B, b, K)\rangle$ may be computed in the same manner as described above for the completely homogeneous population (see 17 in the Methods “Proliferation in neutral case” section) with their respective survival probabilities $\delta _A$ and $\delta _B$, given by (8).

Note within a completely homogeneous population the average probability of reaching the splitting threshold for all-*B* groups is greater than that of all-*A* groups due to the reproduction advantage of *B* individuals (1,2). As a result, a homogeneous population of all-*B* groups can proliferate in some environments where a population consisting of all-*A* groups cannot (Fig. 2B).

**Fig. 3:**
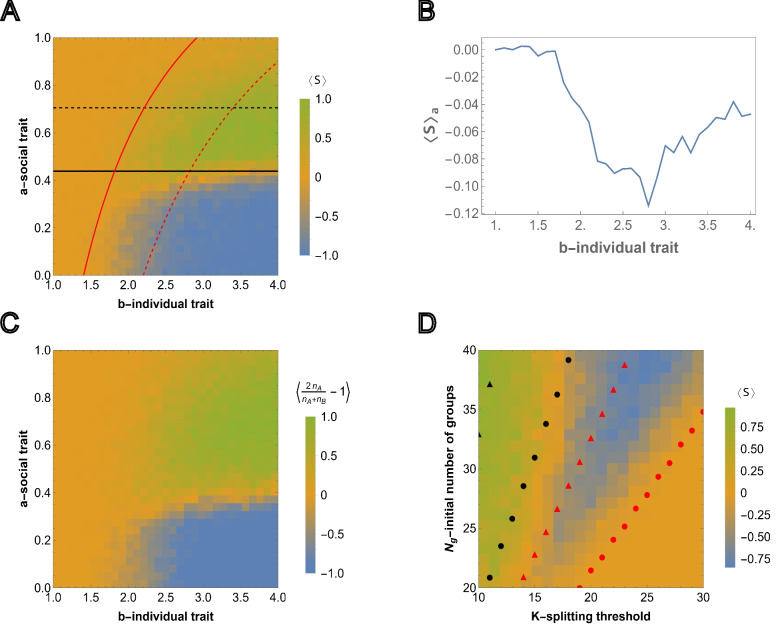
Competition between groups in the case of relative fitness advantage. **A** The results of agent-based simulations are presented for the relative fitness advantage case (7) initialized with equal numbers of homogeneous groups $N_{g,A}=N_{g,B}$. Each cell shows the value of (11) averaged over $M=50$ independent runs, where the sampling is done at $T=3000$. Steps for each cell in the heatmap are chosen with $\Delta a=0.033$ and $\Delta b=0.1$ starting from 0 and 1, respectively. Initial number of homogeneous groups of cooperators is $N_{g,A}=10$. The black and red curves show the lines of $\langle \psi _A\rangle =\frac{1}{2}$ and $\langle \psi _B\rangle =\frac{1}{2}$, respectively. The black and red dashed curves show the threshold values of the social trait and asocial reproductive advantage, $a^*$ and $b^*(a)$, respectively, such that $\langle \psi _A\rangle =\frac{1}{2}$ and $\langle \psi _B\rangle =\frac{1}{2}$. The curves are obtained from (9) and (10), respectively. **B** shows the dependency of the average of $\langle S\rangle$ over all values of the social trait $a\in [0,1]$ for fixed value of $b\in [1,4]$. **C** shows the simulation results for heterogeneous intra-group composition where the initial number of each type of individuals is sampled from *U*(0, *K*/2). The heatmap shows population composition at time $T=5000$, defined by $\langle \frac{2 n_A}{n_A+n_B}-1\rangle$, where $n_A$ and $n_B$ is the total number of cooperators and cheaters in the population. Extinction is assigned an output of 0. The model parameters are $\mu =0.7$, $K=10$, $N_g=20$ and $K_g=70$. **D** Competition outcome for fixed *a* and *b* varying the group splitting threshold $K \in [10,30]$ and initial number of groups $N_g \in [20,40]$, with the initial number of all-*A* groups being equal to the nearest integer-valued lower bound (floor) $N_{g,A} = [0.5 N_{g}]$, for the fixed values of social and asocial traits, *a* and *b*, respectively. The black and red circles show the values of the number of groups satisfying $\langle \psi _A\rangle =\frac{1}{2}$ and $\langle \psi _B\rangle =\frac{1}{2}$ for various *K* in the considered region $N_g \in [20,40]$, respectively. The black and red triangles show the same $\langle \psi _A\rangle =\frac{1}{2}$ and $\langle \psi _B\rangle =\frac{1}{2}$, but obtained for large survival probabilities, (9) and (10), respectively. The model parameters are $\mu =0.7$, $a=0.4$, $b=4$, $K_g=150$ and $T=5000$

### Exploitation of asocial individuals in the case of relative fitness advantage

Competition among social and asocial individuals results in three possible long term outcomes: homogeneous populations of either social or asocial individuals and population extinction. When the environmental conditions (the initial number of groups $N_g$, splitting threshold *K*, and group death probability $\mu$) are favorable for the proliferation of homogeneous populations of the slower-growing, all-*A* groups, extinction rarely occurs and the result of competition depends on the relative strength of the social trait and reproductive advantage of asocial individuals in a straightforward manner. The resulting homogeneous population is asocial if the initial number of asocial groups is large enough and the reproductive advantage is high enough so that, when the total number of groups reaches environmental carrying capacity, $K_g$, if any social individuals remain, the relative fitness advantage provided by the social trait is not strong enough to prevent the stochastic elimination of this small subpopulation.

When the environmental conditions are not favorable for the proliferation of homogeneous populations of all-*A* groups, that is, the environment is harsher, more interesting dynamics are observed. Note that we still consider the environmental carrying capacity, $K_g$, to exceed the survival threshold values (see Fig. 2B). This constraint is relaxed in the next section. Here we also continue to consider the limit where all groups are exclusively composed of one type of individual, *A* or *B*, and extend the analysis to the case of initially heterogeneous groups towards the end of this section. Recall that group proliferation requires that, on average, more than one daughter group survives to split again and the average probabilities of reaching the splitting thresholds for homogeneous groups $\langle \psi _A(\delta _A, K)\rangle$ and $\langle \psi _B(\delta _B, b, K)\rangle$ are given by (17) with their respective survival probabilities $\delta _A$ and $\delta _B$, and transition probabilities $T_{l}^+ = \frac{l}{K}(1-\frac{l}{K})$ and $b T_{l}^+$, for all-*A* and all-*B* groups, respectively. Note that $\langle \psi _{A,B}\rangle$ depend not only on the initial number of groups in total but also on the initial number of all-*A* groups and that $\langle \psi _A\rangle$ is larger in the presence of all-*B* groups. Throughout the figures in the main text, we present the evaluation of initial conditions where there is an equal number of all-*A* and all-*B* groups or an equal number of social and asocial individuals, on average across simulations, in the case of heterogeneous groups.

As in the neutral case, we find the relation between the model parameters that solves $\langle \psi _A\rangle =\langle \psi _B\rangle =\frac{1}{2}$, in the limit of high survival probabilities $\delta _A^*, \delta _B^* \sim 1$. Using (20) and (8), we obtain the following relations for the threshold values of the social trait strength and the reproduction advantage of asocial individuals, $a^*$ and $b^*$, respectively (first (20) is evaluated for $\delta _A^*$ with $b=1$, and then, for $\delta _B^*$ and $b^*$, yielding the relation between *b* and *a*):

9 $$\begin{aligned} & a^*=\frac{(K-1)N_g-2\mu H_{K-1} K^2}{(K-1)N_{g,A}-2\mu H_{K-1} K^2},\end{aligned}$$

10 $$\begin{aligned} & b^*(a)= \frac{2\mu H_{K-1}K^2}{(K-1)(N_g- a N_{g,A})}. \end{aligned}$$

The survival probability of any group depends on the strength of the social trait but not on the asocial reproduction advantage so $a^*$ is independent of *b* but $b^*$ is dependent on *a*. Numerically obtained curves $\langle \psi _A\rangle =\langle \psi _B\rangle =\frac{1}{2}$ and the threshold values (9) and (10) computed in the limit of high survival probability are compared to the results from agent-based simulations in Fig. 3A. The outcome of each simulation run, for various (*a*, *b*) pairs, is defined by

11 $$\begin{aligned} S(T)=\left\{ \begin{array}{ll} 2\frac{N_{g,A}(T)}{N_g(T)}-1, & \text {if}\ N_g(T)\ne 0\\ 0, & \text {if}\ N_g(T)=0 \end{array}\right. \end{aligned}$$

where *T* is sampling time, and $S(T)=1$ and $S(T)=-1$ correspond to the cases where all-*A* groups outcompete all-*B* groups, respectively. The averages of *S*(*T*) are shown for $M=50$ independent simulations $\langle S \rangle = \frac{1}{M} \sum _{\alpha =1}^{M} S_\alpha$. Note that $\langle S\rangle \sim 0$ can result either from extinction or from equal probabilities of all-*A* and all-*B* survival (neutrality with respect to the social trait, corresponding to the yellow region separating green and blue regions in Fig. 3A).

Under these harsher conditions, not favorable for the proliferation of homogeneous populations of all-*A* groups, all three long-term outcomes are observed: extinction, sociality, and asociality. These regions are roughly bounded by the curves $\langle \psi _A\rangle =\frac{1}{2}$ and $\langle \psi _B\rangle =\frac{1}{2}$. When $\langle \psi _B\rangle <\frac{1}{2}$ and $\langle \psi _A\rangle < \frac{1}{2}$, that is, all groups are more likely to die than reach the splitting threshold, the population goes extinct. When $\langle \psi _B\rangle> \frac{1}{2}$ but $\langle \psi _A\rangle <\frac{1}{2}$, that is, all-*A* groups are more likely to die than reach the splitting threshold, only all-*B* groups reach the splitting threshold and the resulting population is asocial. When $\langle \psi _A\rangle> \frac{1}{2}$ both extinction and sociality are possible. When $\langle \psi _B\rangle <\frac{1}{2}$, asocial groups (all-*B*) cannot proliferate and, consequently, the population homogenizes to all-*A* groups before environmental carrying capacity is reached. In this case, the total number of all-*A* groups becomes too small to continue to proliferate and the population goes extinct. Extinction is prevented when $\langle \psi _B\rangle>\frac{1}{2}$. In this case, environmental carrying capacity is reached prior to homogenization and, at the environmental carrying capacity, all-*A* groups out compete all-*B* groups resulting in a stable, social population.

This landscape illustrates the counter-intuitive dependence of the survival of the social groups on the magnitude of the reproduction advantage of the asocial individuals, *b*, introduced above. Indeed, $\langle S \rangle$ shows the average of simulation results for any pair (*a*, *b*), where $a\in [0,1]$ and $b\in [1,4]$. Consider the average of $\langle S \rangle$ over all possible values of $a\in [0,1]$ for a fixed *b* (averaging over rows for a single column of the heatmap matrix), that is $\langle S \rangle _a = \frac{1}{l}\sum _{\Delta a} \langle S(a,b)\rangle$ where *l* is the number of points obtained by dividing the interval [0, 1] in $\Delta a$ steps (Fig. 3B).

Moving from left to right (increasing the magnitude of the b-individual trait) the distribution transitions from a relatively narrow, single peak centered on 0, to a broad, trimodal distribution with peaks centered near −1, 0, and 1. The average taken over all rows indicates the relative weight of these peaks as the b-individual trait increases. The non-monotonic trend displayed in this panel illustrates our principal finding that the presence of noncooperative individuals can promote the persistence of cooperation overall.

For smaller values of *b*, $\langle S \rangle _a\approx 0$, which corresponds to extinction of all groups. $\langle S \rangle _a<0$ means that for any fixed *b*, the intervals of social trait *a* where *A* wins over *B* are smaller compared to the regions where *B* wins over *A*. *A* wins over *B* only within the interval of $a \in [0,1]$, and consequently, $\langle S \rangle _a$ is always near or below 0. $\langle S \rangle _a$ varies non-monotonically with respect to *b*, declining until reaching the range within which both *B* and *A* groups survive, at which point it begins to increase again as the stronger social trait is increasingly supported by the greater reproduction advantage of asocial groups.

From this group-selection perspective, asocial *B* individuals behave as altruists. Indeed, *B* groups “sacrifice” their social-trait to reproduce quickly during the early phase of frequent group elimination so that the threshold group number admitting the fixation of *A* groups can be reached, whereas *B* groups eventually go extinct. It follows that, again counter-intuitively, the survival and proliferation of all-*A* groups is more likely when the initial fraction of these groups is smaller (see Fig. SI2a for the results with a smaller number of all-*A* groups, and Fig. SI2b for the results with the same number of all-*A* groups but a higher splitting threshold *K*). Conversely, from the individual-selection perspective, social *A* individuals behave as altruists “sacrificing” their reproductive advantage to the benefit of the group and asocial *B* individuals behave as “cheaters”. From either perspective, the presence of asocial, noncooperative individuals is not detrimental, but rather, is essential for the persistence of social cooperation.

**Fig. 4:**
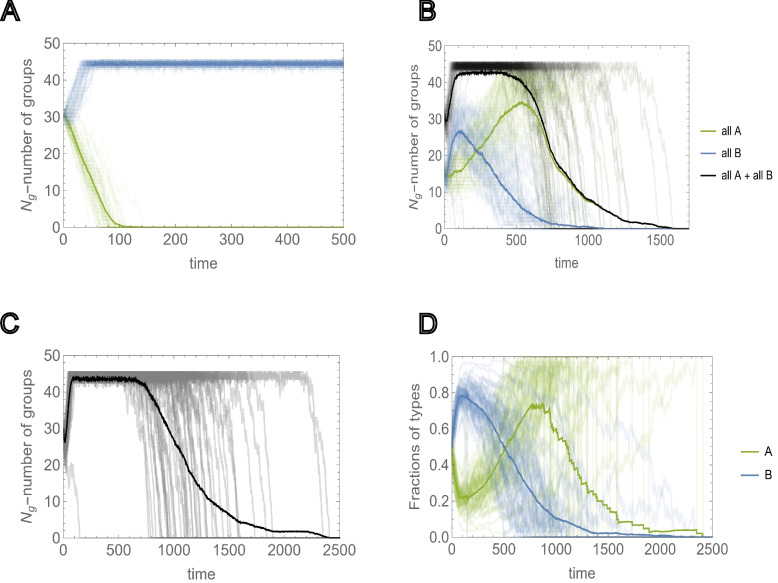
Environmental carrying capacity $K_g$ and extinction due to sociality. Environmental carrying capacity $K_g$ and initial number of groups $N_g$ are chosen such that they allow for the proliferation of homogeneous populations of asocial groups $\langle \psi _B\rangle>\frac{1}{2}$, but not the proliferation of social groups, $\langle \psi _A\rangle <\frac{1}{2}$. **A** Extinction and proliferation of homogeneous populations of social and asocial groups (green and blue curves, respectively) for the same initial number of groups $N_g=30$, obtained in $M=50$ independent runs. **B** Competition between all-*A* and all-*B* homogeneous groups was initialized with equal numbers $N_{g,A}=N_{g,B}$. Green and blue curves show the number of all-*A* and all-*B* homogeneous groups, respectively. Black curves show the time dependency of the total number of groups $N_g(t)$. Thick lines show the behavior of the respective quantities averaged over $M=50$ independent runs. **C** Time dependence of the total number of groups, where the groups are initialized with heterogeneous intra-group composition. The initial number of each type of individual is sampled from *U*(0, *K*/2). **D** Behavior of the fractions of social and asocial individual in the population, *A* and *B*, respectively, during the time of each run of c). Thick lines show the averages of the respective quantities. In all cases, the values of the social trait and individual reproductive advantage, *a* and *b*, are chosen such that in the mixed population, they satisfy (9) and (10), respectively. The model parameters are $\mu =0.5$, $a=0.75$, $b=4$,$K=20$, $N_g=30$ and $K_g=45$

In Fig. 3C, the agent-based simulations are generalized to include heterogeneous groups. In each group, the initial numbers of *A* and *B* individuals are sampled from *U*(0, *K*/2). As before, the color corresponds to the relative number of social and asocial individuals in the total population across all groups at time *T*, that is, we compute $n_A=\sum _{i=1}^{N_g(T)}n_{i, A}$ and $n_B=\sum _{i=1}^{N_g(T)}n_{i, B}$ and display $\frac{2 n_A}{n_A+n_B}-1$ over 50 independent runs with the same model parameters (where simulations resulting in extinction are assigned the value 0). The behavior of the total population in Fig. 3A and in Fig. 3C is very similar indicating that the outcome at the population level is largely independent of the intra-group fixation dynamics.

We further examined the dependence of this landscape on *K* and $N_g$, for fixed *a* and *b*, as shown in Fig. 3D together with the analytical approximations obtained from (9) and (10), respectively. Here, the threshold values of $N_g$ and *K* are found, such that they admit proliferation of all-*A* and all-*B* groups for fixed initial fractions of all-*A* groups in the population, both in the limit of high survival probability, and as the numerically computed curves corresponding to $\langle \psi _A\rangle =\frac{1}{2}$ and $\langle \psi _B\rangle =\frac{1}{2}$. For increasing *K*, the minimum initial $N_g$ required for group proliferation increases, and this threshold for social individuals is higher than that for asocial individuals. The numerical results approximate a lower bound for $N_g$ with respect to *K* and, despite the required simplification, the analytical approximations coincide with the appropriate regions of the phase space.

### Propensity for sociality results in extinction in resource-limited environments

In the previous section, we demonstrated that, within a multilevel selection framework, counterintuitively, the presence of asocial, noncooperative individuals is not detrimental, but on the contrary, is essential for the persistence of social cooperation. We additionally demonstrated that, when environmental conditions do not support the proliferation of a homogeneous population of social groups, but support the proliferation of a homogeneous asocial population, social groups can outcompete asocial groups, resulting in extinction of the entire population. So far, we have assumed that the environmental carrying capacity, $K_g$, is larger than the number of groups required for the survival of a homogeneous social population (see Fig. 2B) and, consequently, when $b>1$ homogeneous asocial populations also survive. That is, for given values of $\mu$ and *K*, $K_g$ is above the threshold value defined by (5) (substituting $N_g$ by $K_g$, and $b=1$). Under this assumption, as long as environmental carrying capacity is reached, extinction is prevented.

We now consider the case where $K_g$ is smaller than the threshold value that supports the survival of a homogeneous population of all-*A* groups, but allows the survival of a homogeneous population of all-*B* groups, $\langle \psi _A(\delta (\mu ,K_g),K) \rangle {<}\frac{1}{2}$ and $\langle \psi _B(\delta (\mu ,K_g),K) \rangle {>}\frac{1}{2}$, respectively (see Fig. 4A). We assume that the initial number of groups $N_g<K_g$ is such that $\langle \psi _B(\delta (\mu ,N_g),K) \rangle {>}\frac{1}{2}$. In this setting, if a sufficient number of all-A groups is present, the population will go extinct (Fig. 4B, C). Figure 4B illustrates the mixed homogeneous group case.

Here, the entire population quickly reaches the environmental carrying capacity of the environment, primarily via proliferation of all-*B* groups. The initial proliferation of all-*B* groups increases the survival probability for all-*A* groups, leading to the subsequent decline of *B* groups, and eventually, to the collapse of the entire population. The same behavior is observed for heterogeneous group composition (Fig. 4C, D). Here, the fraction of *B* individuals in the population, $\frac{\sum _{j=1}^{N_g(t)} n_{j,B}}{\sum _{j=1}^{N_g(t)} n_{j,B}+n_{j,A}}$, increases initially due to the reproduction advantage of *B*-dominated groups. These groups reach the splitting threshold faster than *A*-dominated groups; however, *A*-dominated groups outcompete in the long run, eventually resulting in the homogenization and subsequent extinction of the entire population.

### Absolute fitness advantage of the social trait

In the previous sections, we assumed that sociality manifested at the group level, providing a relative fitness advantage. In this case, groups with a greater *A* fraction are less likely to be eliminated than groups with a smaller *A* fraction, but the overall probability of group death for a homogeneous *A* population and a homogeneous *B* population is the same. Here, we consider an alternative functional form for the social trait such that the fitness advantage provided by *A* individuals within the group is absolute, that is, independent of the composition of the population, and can affect the total probability of any group death. In this case, the probability that group *j* will die at time *t* is:

12 $$\begin{aligned} P_{j,death}(t)=\frac{\mu }{N_g(t)} (1-a\frac{n_{j,A}(t)}{n_{j,A}(t)+n_{j,B}(t)}), \end{aligned}$$

The first term in (12) is the probability that group *j* is selected at random among all $N_g(t)$ groups. The second term is the probability of death once the group is selected. As described above, in contrast to the previous case, $\sum _{j}P_{j,death}\le \mu$. Note that $P_{j}$ is independent of the composition of other groups, representing frequency-independence at the group level.

**Fig. 5:**
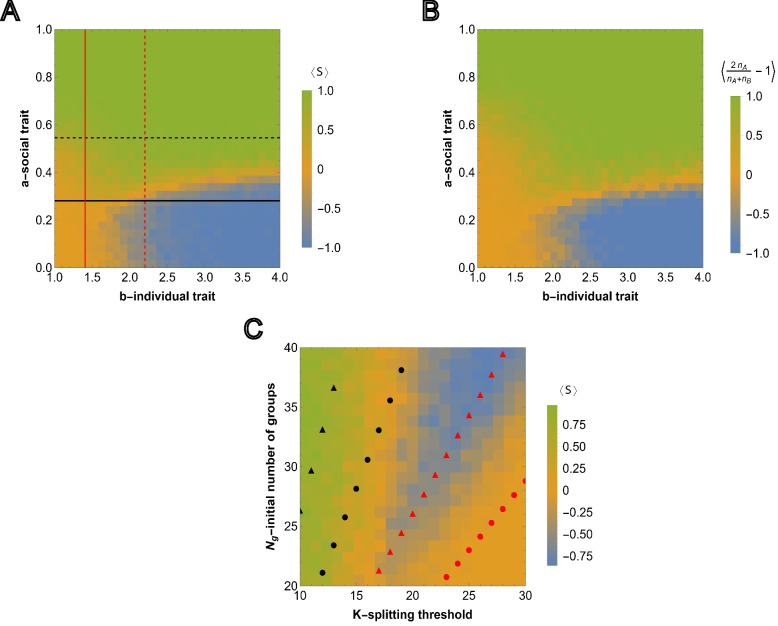
Competition outcome between social and asocial groups under absolute fitness advantage. **A** Results of agent-based simulations are presented for absolute fitness advantage (12) for homogeneous groups $N_{g,A}=N_{g,B}$. Steps for each cell in the heatmap are the same as in Fig. 3A. The red and black solid lines show the values of $a^*$ and $b^*$, such that $\langle \psi _A\rangle =\langle \psi _B\rangle =\frac{1}{2}$. The dashed lines show $a^*$ and $b^*$, obtained from (14) and (15), corresponding to $\langle \psi _A\rangle =\langle \psi _B\rangle =\frac{1}{2}$ in the limit of large survival probabilities. **B** Counterpart of Fig. 3C is presented for the case of absolute fitness advantage. The model parameters are the same as in Fig. 3A. **C** Counterpart of Fig. 3D for the absolute fitness advantage case with the same model parameters

The survival probabilities of all-*A* and all-*B* groups are independent of population composition:

13 $$\begin{aligned} & \delta _A=1-\frac{\mu (1-a)}{N_g},\quad \delta _B=1-\frac{\mu }{N_g}, \end{aligned}$$

The survival probability of all-*B* groups is identical to the neutral case whereas the survival probability of all-*A* groups is rescaled with respect to the group death probability $\mu \rightarrow \mu (1-a)$.

The threshold values of *K* and $N_g$ admitting proliferation of all-*A* and all-*B* populations depend on the magnitude of the social trait *a* and the asocial reproduction advantage *b*. In this case, social groups outcompete asocial groups whenever $\langle \psi _A\rangle>\frac{1}{2}$, but lose otherwise. Extinction of all groups occurs when $\langle \psi _A\rangle <\frac{1}{2}$ and $\langle \psi _B\rangle <\frac{1}{2}$. In contrast to the relative advantage case, here, $\langle \psi _A\rangle =\frac{1}{2}$ and $\langle \psi _B\rangle =\frac{1}{2}$ yield constant values for the magnitude of the social trait and reproduction advantage, *a* and *b*, respectively, due to the independence of the absolute fitness advantage with respect to the population composition.

The threshold values of the social trait and individual reproductive advantage in the limit of high survival probabilities, $a^*$ and $b^*$, that solve $\langle \psi _A(\delta _A, K)\rangle =\langle \psi _B(\delta _B, K, b)\rangle =\frac{1}{2}$ are:

14 $$\begin{aligned} & a^*=1-\frac{(K-1)N_g}{2\mu H_{K-1}K^2},\end{aligned}$$

15 $$\begin{aligned} & b^*=\frac{2\mu H_{K-1}K^2}{(K-1)N_g}. \end{aligned}$$

By construction, $a\in [0,1]$ and $b\in [1,4]$. The dashed lines in Fig. 5A show $a^*$ and $b^*$ in the limit of high survival probabilities, obtained from (14) and (15), respectively. The observed trends are qualitatively similar to those obtained with the relative advantage of the social trait (Fig. 3), with a similar agreement between the analytical approximations and the simulation. In Fig. SI3, the counterparts of Fig. 5 are shown, for a smaller initial fraction of homogeneous social groups, $N_{g, A}$, and splitting thresholds $K=10$ and $K=15$.

In contrast to the relative fitness case, when sociality confers an absolute fitness advantage that reduces the overall death probability, the persistence of social groups does not depend on the presence of asocial groups. Contrasting Fig. 5 with Fig. 3, the phase portrait is simpler in the case of absolute fitness advantage of the social trait than in the case of relative fitness advantage. For absolute fitness advantage, extinction is less prominent and can be prevented by increasing the strength of the social trait independent of the magnitude of the asocial reproductive advantage. Notably, in terms of the three possible long-term outcomes — sociality, asociality, and extinction — the phase portraits differ specifically with respect to extinction. By construction, both models have the same fixation properties when extinction is rare and our results for the relative fitness advantage case could not have been obtained within a framework where the number of individuals or groups is fixed (see Discussion).

### Biofilm formation as an example of cheater-cooperator co-evolution

In the previous sections, we presented a general model of multilevel selection demonstrating counterintuitive dynamics in which emergence and persistence of an altruistic trait can be facilitated by or even depend on the presence of cheaters (asocial individuals).

As presented at the beginning of the Model section, our general model relies on three principal assumptions: the utility of a multilevel selection modeling framework; selection at the individual level is frequency independent; and selection at the group level is frequency dependent. Here we introduce a specific biophysical model, for biofilm formation, that exhibits the behavior central to our main findings while relaxing the conditions that support the last of these assumptions. Our biofilm model is also a multilevel selection framework and selection at the individual level is also frequency-independent, through the production of extracellular matrix proteins as public goods.

In contrast, within our theoretical model, frequency-dependent selection at the group level is introduced through the implementation of a constant rate of group death, independent of the total number of groups. This idealized model is the limiting case of a much more general condition, where the fraction of groups susceptible to death grows slower than the total number of groups and, consequently, the probability of group death declines with the total number of groups. In the biofilm model described below, death only occurs at the biofilm surface, which grows proportionally to the square root of the total population size, without requiring the much stronger assumption that the rate of group death is constant.

We note that we do not claim that our biofilm simulation incorporates all the necessary details to predict the behavior of specific, individual biofilms. Biofilms are complex systems shaped by diverse factors not included in the simulation below [63]. For a recent review on biofilm modeling, both computational and experimental, please see the cited reference [64]. Moreover, as pointed out in the Introduction, we invoke the biofilm as just one of many possible spatially-structured biological systems that can be coarsely divided into two populations: the bulk and and the interface subject to higher rates of mortality. Other examples include, but are not limited to, populations of grazing animals [65] and solid tumors [66].

In our simulation, biofilms are composed of two cell types, social individuals producing extracellular matrix (ECM) proteins which bind neighbors together [57] in exchange for a growth cost and asocial individuals which do not make ECM but can use it. Cells experience a repulsive pseudo-force acting at short distances and an attractive force at long distances. Biofilm growth is simplified to be logistic[67], constrained by an environmental carrying capacity of 500 cells. ECM production is simulated as a 100-fold increase in the attractive force constant and a 5-fold decrease in the rate of cell division. Cells are also attracted to the substrate and subject to random motion. We note that while the substrate is represented with a lattice model, the coordinates of individual cells are continuous (up to discretization). Additionally, the distances between substrate lattice sites is small relative to most intra-cell distances.

The predation interface is composed of cells at the top of the biofilm (those which have no neighbors directly above them). Each biofilm, representing the whole population, is organized into dynamic local spatial groups, reflecting the complex spatial structure observed within real biofilms [68].

ECM production provides a relative fitness advantage to local groups of neighboring cells by decreasing the probability that cells from that group will migrate into the predation interface. It is an altruistic trait at the individual level as asocial individuals within that local group benefit from the stronger attractive pseudo-force without paying the growth cost. Consequently, in this specific system, as shown above more generally, asocial individuals are cheaters at the individual level but become altruists at the group level (see Methods for details).

**Fig. 6:**
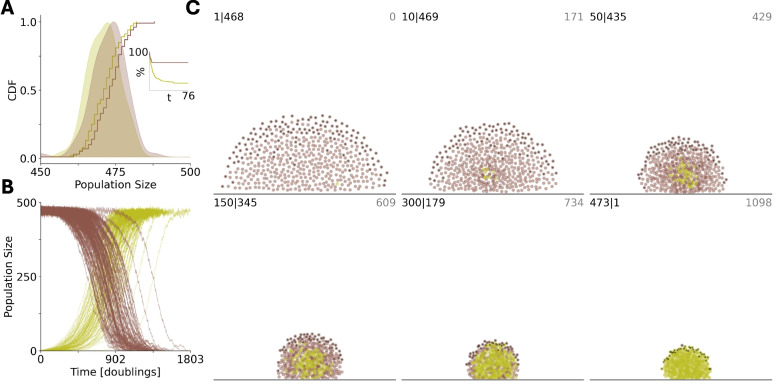
Social biofilm architects (green) benefit from asocial neighbors (brown). **A** homogeneous populations are of equal size at steady-state (100 trials shown). Inset. The percentage of seeding simulations which have resisted extinction vs time. **B** at time 0, a single individual acquires a mutation conferring the social phenotype in a homogeneous biofilm previously at steady-state. The mutation fixes with high probability (99 of 100 trials shown). **C** timelapse of individual fixation simulation. number social| number asocial, in top left; time in top right of each panel. Individuals at the predation interface are identified in black. **A**/**B**/**C**. Time is measured in doubling times for asocial individuals far from the environmental carrying capacity

At steady-state, within this parameter regime, homogeneous biofilms of ECM-producers and non-producers are approximately the same size (Fig. 6A) and, consequently, ECM production is a neutral trait at the whole- biofilm level. In contrast, in the context of colony propagation via seeding [69] by which a single individual anchors to the substrate to produce a new biofilm, ECM production is a strongly deleterious trait. The success rate for biofilm seeding is approximately 4-fold higher for the asocial non-producers (72/100 trials for non-producers; 19/100 trials for ECM-producers, see Fig. 6A inset). Due to the associated relative fitness advantage, mutations conferring ECM production fix with high probability within homogeneous biofilms of non-producers (99/100 trials, see Fig. 6B). Figure 6C illustrates a timelapse of an example fixation simulation. It follows that the presence of cheaters facilitates the emergence and persistence of altruists in this system.

## Discussion

In this work, we consider a two-level (individual and group) selection scenario in which groups are composed of a mixture of asocial and social individuals. We demonstrate that from the individual-selection perspective, social individuals behave as altruists and asocial individuals behave as cheaters. However, although the presence of cheaters is typically associated with a negative impact on the survival and growth of social cooperators [14, 18, 60], we demonstrate the counter-intuitive phenomenon whereby, in the context of multilevel selection, the emergence of cheaters can promote and even can be essential for the long-term survival of cooperators. This is consistent with seminal work on the division of labor in multiple Public Good Games where behavior classically regarded as “cheating” can increase group fitness [70]. Our model is simple, but generalizable, and can be further developed to represent a wide variety of biological systems while still admitting several useful analytical results.

We first considered a social trait that provides a relative fitness advantage at the group level, that is, groups containing a higher proportion of individuals with the social trait (cooperators) are relatively more likely to survive than other groups. The cost of this social trait is paid at the individual level so that individuals lacking the social trait (cheaters, at the individual level) have a reproductive advantage. The reproductive advantage of the cheaters is independent of the composition of the population, as opposed to the frequency-dependent fitness more commonly assumed in evolutionary game theory [28, 48, 60].

Under these conditions, we observed three related counter- intuitive phenomena. (1) Seeding the initial population with a greater number of cheaters that do not carry the social trait tends to increase the probability that the social trait will eventually be fixed. (2) The greater the strength of the social trait, the greater the reproductive advantage of cheaters that is required to admit the proliferation of cooperator groups. (3) Cheaters can survive in environments with lower environmental carrying capacities than cooperators thanks to their reproductive advantage; however, once environmental carrying capacity is reached, groups of cooperators outcompete cheaters. Consequently, the population becomes dominated by social groups, and subsequently, goes extinct.

The beneficial effect of asocial individuals on the survival of sociality is a consequence of multilevel selection whereby asocial individuals effectively behave as altruists at the group level. This occurs because the survival of the population — and hence the survival of social individuals — requires that the total number of groups exceeds a certain threshold, and that critical population size is reached due to the fast proliferation of asocial individuals.

We then considered an alternative model of a social trait conferring an absolute fitness advantage. In this case, the survival of a group is defined only by its composition and does not depend on the composition of other groups in the population. In this regime proliferation of social individuals does not depend on the initial presence of asocial individuals. Exploration of this model yielded an additional methodological insight. Both models analyzed, with a relative or absolute fitness advantage operating at the group level, result in the same classical birth-death process in the limit of infrequent group death and fixed group number, depending only on the strength of the social trait. In other words, the outcomes of the two models differ only with respect to extinction, and whenever the social trait is fixed in one case, it will be fixed in the other case as well, barring extinction. It follows that evaluation of many existing models of multi-level selection [48, 50, 51, 54] where the number of individuals or groups is fixed, is inadequate to identify conditions sufficient for the evolution of social traits.

In both cases, we find that lowering the group splitting threshold and increasing the initial number of groups benefits social individuals [15, 48, 51]. In homogeneous populations of social and asocial individuals with the same initial number of groups, cooperators can only survive when the splitting threshold is relatively lower (resulting in smaller groups) because of their reproductive disadvantage. Similarly, for the same splitting threshold, asocial individuals are able to proliferate with a smaller initial number of groups than social individuals. This also holds for heterogeneous populations with either relative or absolute fitness advantages provided to groups by the social individuals.

We discussed one principal limitation of our theoretical framework, the strong assumption of a constant rate of group death, and how this is the limiting case of a much more general condition that naturally emerges from spatial structure. Another limitation of our approach is the assumption that the growth cost of the social trait is time-invariant. More generally, this cost can be determined by the environment or the population structure. For example, if the growth cost comes from the production of a public good the synthesis of which is automatically triggered by its low extracellular concentration, high extracellular concentration of that public good results in decreased growth cost. Under these conditions, the growth cost depends not only on global environmental perturbations but also on proximity to other social individuals.

We also assume that individuals cannot change status: each individual is either always social or always asocial. More generally, sociality can also be modified at the individual level in response to environmental perturbation or population structure. For example, if social individuals are capable of recognizing other social individuals, it may be possible to participate in the production of public goods only when the local population is reciprocating. Allowing the cost of the social trait to be environmentally or time dependent would also support more diverse spatially-mediated evolutionary strategies.

Additionally, the results presented in this work correspond to homogeneous populations of social or asocial individuals (at equilibrium) without the possibility of coexistence; however, coexistence is observed in some biological systems. For example, in the classic model system of snowflake yeast, where sucrose hydrolysis is a cooperative trait, both social and asocial traits are mutually invadable [71]. These dynamics are attributable to the saturating effect of the public goods produced by the cooperators in this system. While the benefits of the social trait are also saturating within our framework, (7), this feature alone is insufficient to maintain coexistence under the conditions we study and the introduction of spatially or time dependent parameters which could recover coexistence is a topic for future investigation.

## Conclusions

In conclusion, we propose a simple, generalizable framework to explore evolution of cooperation that admits several useful analytical results. We validate key findings with an agent-based model of a specific system, a biofilm, where the production of extracellular matrix proteins as public goods and the resulting spatial structure that leads to the restriction of deaths to the bioflim surface, introduces frequency-independent selection at the individual level and frequency-dependent selection at the group level. This is just one example of the diversity of biophysical mechanisms that can produce real ecological structures relevant for our modeling framework, for example, toxin production specifically targeting non-kin individuals.

We demonstrate, counterintuitively, that across a broad range of conditions, the presence of cheaters is essential for the proliferation of cooperators such that introduction of stronger social traits is insufficient for cooperation to evolve. On the contrary, stronger cooperators require stronger cheaters. Conceivably, our approach can be extended to model host-parasite coevolution, potentially, yielding a better understanding of the role of parasites in the evolution of life by multilevel selection [13]. More generally, these results stem from the frustration between the selective factors operating at different levels of organization (individual and group) which seems to underpin the evolution of complexity [72, 73].

## Methods

### Proliferation in neutral case

For any initial state, we denote the probability of reaching the absorbing state *K* by $\psi _{n}$. Noting that the probabilities at the boundaries $\psi _0=0$, corresponding to group elimination, and $\psi _{K}=1$, corresponding to the splitting threshold, are known, the remaining values of $\psi _n$ may be found by solving the following recursive equation [74]

16 $$\begin{aligned} \psi _{n}= \delta T_n^{+}\psi _{n+1} + \delta (1-T_n^{+}) \psi _{n} + (1-\delta ) \psi _{0} \end{aligned}$$

which can be understood as follows. Consider a group in state *n* at time *t*. If it survives until time $t+1$, with probability $\delta$, it can move to state $n+1$ with probability $T_n^{+}=b \frac{n}{K}(1-\frac{n}{K})$ or stay in state *n* with probability $1-T_n^{+}$. If it attains state $n+1$, it reaches the splitting threshold with probability $\psi _{n+1}$ (first term) and otherwise $\psi _{n}$ (second term). The last term, corresponding to group elimination, is 0 and is included for completeness. Incorporation of individual death within groups results in a recurrence relation similar to (16) with an additional term describing transition from $n\rightarrow n-1$ (see Additional File 1).

The solution to (16) is

17 $$\begin{aligned} \psi _{n}= \prod _{l=n}^{K-1} \frac{\delta T_{l}^+}{1-\delta +\delta T_{l}^+} \end{aligned}$$

Note that, in the absence of group death, corresponding to $\delta =1$, all groups eventually reach the splitting threshold, $\psi _n=1$. Denoting the average of (17) over all possible initial states $\langle \psi (\delta , K) \rangle =\frac{1}{K-1}\sum _{n=1}^{K-1}\psi _n$, the expectation that for the given values of $\mu , N_g$ and *K*, groups will proliferate, satisfies the inequality:

18 $$\begin{aligned} \langle \psi (\delta ,b, K) \rangle>1/2 \end{aligned}$$

Thus, a group will proliferate if it is more likely to reach the splitting threshold than to die. The average probability of reaching the threshold increases with the increasing number of groups $\frac{\partial \langle \psi \rangle }{\partial N_g}>0$, and decreases with increasing death probability $\frac{\partial \langle \psi \rangle }{\partial \mu }<0$ and with increasing splitting threshold value $\frac{\partial \langle \psi \rangle }{\partial K}<0$. In the limit of high survival probability, $\delta \sim 1$, the average probability of reaching the splitting threshold is given by (see Additional File 1 for derivation):

19 $$\begin{aligned} \langle \psi (\delta ,b, K)\rangle =1-(1-\delta )H_{K-1}\frac{K^2}{b(K-1)} \end{aligned}$$

where $H_{n}$ is the harmonic number. From (19), one can also compute the threshold value for $\delta$ satisfying $\langle \psi (\delta ^*,b, K) \rangle =1/2$, that is, the value specifying when it is equally likely, on average, to reach the splitting threshold or die:

20 $$\begin{aligned} \delta ^*=1-\frac{K-1}{2 H_{K-1} K^2} b \end{aligned}$$

Substituting the expression for survival probability, $\delta = 1- \frac{\mu }{N_g}$, into (20), we find the relation among the model parameters (5).

### Relative and absolute fitness cases for $\mu \ll 1$

Under the $\mu \ll 1$ limit, let us assume that a population of homogeneous groups has reached the environmental carrying capacity $K_g$, such that $N_{g, A}$ groups are composed of only *A* individuals and $K_g-N_{g, A}$ of only *B* individuals. Indeed, for $\mu \ll 1$, all initially heterogeneous groups will homogenize first before fixation of any trait in the population. Let us further assume that each group in the population reaches the splitting threshold *K* before the next reproduction event, given that $\mu \ll 1$.This assumption also implies that group reproduction is trait-independent. Although *B* groups will, on average, reach the splitting threshold faster than *A* groups, at the environmental carrying capacity, when group death is infrequent, all groups can be presumed to be at the splitting threshold at the time of any group death. These considerations yield the transition probabilities for the relative fitness advantage case (7):

21 $$\begin{aligned} & \tilde{T}(N_{g,A} \rightarrow N_{g,A}-1)=\mu \frac{K_g-N_{g,A}}{K_g}\frac{N_{g,A}(1-a)}{N_{g,A}(1-a)+K_g-N_{g,A}} \nonumber \\ & \tilde{T}(N_{g,A} \rightarrow N_{g,A}+1)=\mu \frac{N_{g,A}}{K_g}\frac{K_g-N_{g,A}}{N_{g,A}(1-a)+K_g-N_{g,A}}, \end{aligned}$$

where, in the first equation, the first ratio is the probability that a *B* group splits at the time of group death and the second ratio is the probability that an *A* group was eliminated. Note that the group death probability $\mu$ impacts the rate of fixation but not the final state, which is determined by the ratio of the transition probabilities, $1-a$.

In the same limit, the absolute fitness advantage, (12), yields the following transition probabilities

22 $$\begin{aligned} & \hat{T}(N_{g,A} \rightarrow N_{g,A}-1)=\mu (1-a) \frac{N_{g,A}}{K_g}\frac{K_g-N_{g,A}}{K_g}, \nonumber \\ & \hat{T}(N_{g,A} \rightarrow N_{g,A}+1)=\mu \frac{N_{g,A}}{K_g}\frac{K_g-N_{g,A}}{K_g}, \end{aligned}$$

resulting in the same fixation properties dependent only on the ratio, $1-a$.

### Biofilm simulation

Biofilms reside on a substrate consisting of 400 anchor points uniformly distributed on a grid approximately twice the length of the footprint of the broadest biofilm to mitigate boundary effects. Biofilm dynamics may be subdivided into three categories: cell motion, cell division, and cell removal (death/predation). Cells move at a fixed speed of one gridpoint per timestep in a direction determined by pseudo-forces acting on them by other cells and the substrate as well as a random term. The pseudo-force between cells *p* and *q* is governed by the expression: $\frac{H}{d\left( p,q\right) ^{6}}-\frac{G\left( p\right) +G\left( q\right) }{2d\left( p,q\right) ^{2}}$ where *d*(*p*, *q*) is the distance separating the pair, *H* is the repulsive pseudo-force constant, and *G* is the attractive pseudo-force constant. Across all simulations shown, $H=1$ (arbitrary) and *G* were selected to satisfy the condition: $\frac{H}{D^{6}}-\frac{G}{D^{2}}=0$, for a pair of asocial individuals, where *D* is the distance between adjacent substrate grid points. For social individuals, *G* is 100 fold larger. The pseudo-force between a single cell and each substrate anchor is of the same form where $\frac{G\left( p\right) +G\left( q\right) }{2}$ is substituted by $\frac{G}{2}$. After calculating the pseudo-forces acting on each cell, the unit vector in the same direction is computed and the weighted average of it and a unit vector in a random direction is obtained: $\left( 1-F\right) u_{force}+F_{u_{rand}}$ where the parameter, *F*, defines the degree to which cell motion is randomized. Across all simulations shown, $F=0.5$. Cell positions are then updated according to movement in the specified direction and a reflective boundary condition on the substrate is imposed. Finally, a small random displacement is added to each cell position to ensure uniqueness and prevent division by zero.

After computing cell motion, cell division is implemented. Cell division depends only on the social phenotype and not on location within the biofilm or history of prior division. For each cell type, social and asocial individuals, a poisson pseudo random number is drawn with expected value: $n_{BF}r(1-N_{BF}/K_{BF})dt$ where $n_{BF}$ is the number of cells of the given type, *r* is the rate of cell division far from the environmental carrying capacity, $N_{BF}$ is the total number of cells in the biofilm, $K_{BF}$ is the environmental carrying capacity, and *dt* is the timestep. Across all simulations shown, *r* for asocial individuals is *ln*(2) (and the doubling time far from environmental carrying capacity is 1) and *r* for social individuals is fivefold less; $K_{BF}=500$; and $dt=0.01$ (so that asocial individuals move for 100 time steps on average prior to dividing when far from environmental carrying capacity). The selected quantity of cells of each type (or, if exceeding the total number, all cells of that type) are duplicated and a small random displacement is added to each cell position.

Following division, removal is implemented as follows. The cells farthest from the substrate along the vertical axis within any grid window are identified. The predation interface is composed of these cells. A poisson pseudo random number is drawn with expected value: *mLdt* where *m* is the number of cells within the predation interface, *L* is the rate of cell death, and *dt* is the timestep. Across all simulations shown, $L=0.1$.

Three types of simulations were performed: the evaluation of steady-state behavior, biofilm seeding, and the fixation of a mutation conferring the social phenotype. To evaluate steady-state behavior, homogeneous populations of $K_B$ cells of either type were initialized at uniformly distributed random positions within a square at the center of the substrate grid, with width 5 percent the length of the grid. Cell positions were updated for 1000 timesteps without random motion ($F=0$), division, or removal. The full simulation then proceeded for 20000 timesteps and the final state was observed. To simulate biofilm seeding, the same procedure was followed beginning with a single cell and stopping when the population exceeded 300 cells. To simulate fixation, the biofilm was initialized according to cell positions obtained from a steady-state evaluation simulation for asocial individuals (randomly selected out of 100 trials performed). A single cell was then re-labeled as a social individual (randomly selected) and dynamics were simulated until the biofilm homogenized.

## Supplementary Information

Additional file 1. Section I, Incorporating individual death into the model. Section II, Threshold relation between model parameters in high survival regime - derivation of equation five in the main text. Section II, Supplementary figures. Figure S1, Survival of groups for different values of model parameters. Figure S2, Competition outcome between groups of social and asocial individuals in the case of relative fitness advantage. Figure S3, Competition outcome between groups of social and asocial individuals in the case of absolute fitness advantage.

## Data Availability

All data generated or analyzed during this study, limited to the code used to conduct the simulations, are available here: https://zenodo.org/records/17803218 [75].
