# Supplementary material for: The key role of cheaters in the persistence of cooperation
Source: BMC Biol. 2026 Jan 3;24:29. doi: 10.1186/s12915-025-02492-5 (PMC12866054; doi:10.1186/s12915-025-02492-5)
Supplement: Supplementary file 1 — Additional file 1. Section I, Incorporating individual death into the model. Section II, Threshold relation between model parameters in high survival regime - derivation of equation five in the main text. Section II, Supplementary figures. Figure S1, Survival of groups for different values of model parameters. Figure S2, Competition outcome between groups of social and asocial individuals in the case of relative fitness advantage. Figure S3, Competition outcome between groups of social and asocial individuals in the case of absolute fitness advantage. [file 12915_2025_2492_MOESM1_ESM.pdf]

Supplementary Information

I. INCORPORATING INDIVIDUAL DEATH INTO THE MODEL

In the main text, we do not include individual death in the model. Here, we will explore the effect of including this type of event, whereby an individual dies and its site within its group is replaced by resources.

Let us consider again the neutral case whereby $b = 1$ and $a = 0$, that is, there is no difference between social and asocial individuals. Denoting the number of individual in a group by n, in the given time step, it is possible to have either $n \to n+1$, $n \to n-1$ and $n \to n$. To impose the normalization condition on the transition probabilities, we introduce the parameter $\nu \in [0,1]$ that controls birth vs death probabilities.

For the transition probabilities, we obtain the following expressions:

$$\hat{T}_n^+ \equiv T(n \to n+1) = \nu \frac{n}{K}(1 - \frac{n}{K}), \tag{1}$$

$$\hat{T}_n^- \equiv T(n \to n-1) = (1-\nu)\frac{n}{K}. \tag{2}$$

Note that $\hat{T}_n^+ + \hat{T}_n^- \leq 1$. The survival probability of the group, with respect to external forces, δ will not change due to individual death. However, the probability to reach the splitting threshold starting from n, ψ_n, will change. In this case, the recurrence relation governing ψ_n has the following form

$$\psi_n = \delta \hat{T}_n^+ \psi_{n+1} + \delta \hat{T}_n^- \psi_{n-1} + \delta(1 - \hat{T}_n^+ - \hat{T}_n^-)\psi_n + (1-\delta)\psi_0 \tag{3}$$

As in the main text, the first term describes the reproduction of an individual within the group, given that the group survives. The second term describes the death of an individual given that the group survives. The third term corresponds to the case of no reproduction or death within the surviving group. The last term corresponds to the death of the group.

What follows from (3), is that the results obtained in the main text will be affected quantitatively, and will take a place in different regions of model parameters where reaching the splitting threshold is more likely than the death of a group on average, in general. Therefore, for the sake of simplicity, we proceed by neglecting the individual death event within groups, and direct competition of the individuals within group. The last effect could be incorporated in the model in a similar way.

II. THRESHOLD RELATION BETWEEN MODEL PARAMETERS IN HIGH SURVIVAL REGIME: DERIVATION OF EQ. (5) IN THE MAIN TEXT.

We start from $\psi_n = \prod_{l=n}^{K-1} \frac{\delta T_l^+}{1-\delta+\delta T_l^+}$ and assume $b = 1$. Observe that for $1 - \delta \approx 0$ each term in the product can be written as

$$\frac{\delta T_l^+}{1-\delta+\delta T_l^+} = 1 - \frac{1}{T_l^+}(1-\delta) + O((1-\delta)^2) \equiv 1 - c_l x \tag{4}$$

where we expand the left-hand side at $\delta \approx 1$. We have denoted $c_l = \frac{1}{T_l^+}$ and $x = 1 - \delta$. Then, each ψ_n can be approximated as follows

$$\psi_n = \prod_{l=n}^{K-1}(1 - c_l x) \approx 1 - x\sum_{l=n}^{K-1} c_l \tag{5}$$

where we neglect the terms $O(x^2)$. The sum involved in (5) is equal to

$$\begin{aligned}\sum_{l=n}^{K-1} c_l &= \sum_{l=n}^{K-1} \frac{1}{T_l^+} = K\sum_{l=n}^{K-1}\frac{K}{l(K-l)} = K(\sum_{l=n}^{K-1}\frac{1}{l} + \sum_{l=n}^{K-1}\frac{1}{(K-l)}) = \\ &= K(H_{K-1} - H_{n-1} + H_{K-n}) = K(H_{K-1} - H_n + H_{K-n} + \frac{1}{n})\end{aligned}$$

where $H_m = \sum_{j=1}^{m} \frac{1}{j}$ is the mth harmonic number. In the first line, we interchanged the variable $j \equiv K - l$ in the second sum. In the second line, we used the identity $H_m = H_{m-1} + \frac{1}{m}$. Now, we use the definition of the average probability to reach the splitting threshold, that is

$$\begin{aligned} \langle \psi \rangle &= \frac{1}{K-1} \sum_{n=1}^{K-1} \psi_n = \frac{1}{K-1} \sum_{n=1}^{K-1} \left(1 - xK(H_{K-1} - H_n + H_{K-n} + \frac{1}{n})\right) = \\ &= 1 - x\frac{K}{K-1}(\sum_{n=1}^{K-1} H_{K-1} + \sum_{n=1}^{K-1} \frac{1}{n}) = 1 - x\frac{K^2}{K-1} H_{K-1}. \end{aligned} \quad (6)$$

where we have used the fact that $\sum_{n=1}^{K-1} H_{K-n} - \sum_{n=1}^{K-1} H_n = 0$. Finally, from $\langle \psi \rangle = \frac{1}{2}$ and $x = 1 - \delta$, we find that

$$\delta^* = 1 - \frac{K-1}{2H_{K-1}K^2} \quad (7)$$

In (4) we neglect the terms of $O((1-\delta)^2) \equiv O(x^2)$. The higher order terms in (4) are given by the following expression

$$O(x^2) = \frac{1 - T_l^+}{{T_l^+}^2} x^2 \sum_{i=0}^{\infty} (-1)^i (\frac{1 - T_l^+}{T_l^+} x)^i \quad (8)$$

Now, (8) converges if $x < \frac{T_l^+}{1-T_l^+}$. If the last condition holds, then:

$$O(x^2) = \frac{1 - T_l^+}{{T_l^+}^2} x^2 \frac{1}{1 + \frac{1-T_l^+}{T_l^+} x} \quad (9)$$

which is positive and bounded again if $x < \frac{T_l^+}{1-T_l^+}$, since $T_l^+ < 1$. Note, that if $x < \frac{T_l^+}{1-T_l^+}$ holds for $l = 1$ and $l = K - 1$, then it holds for all $l = 2, ..K - 2$.

Let us check that x^* obtained from (7) satisfy this condition. We obtain

$$\frac{K-1}{2H_{K-1}K^2} < \frac{K-1}{K^2} \frac{1}{1 - \frac{K-1}{K^2}} \quad (10)$$

from which, we obtain that x^* satisfies the assumption if

$$2H_{K-1} > 1 - \frac{K-1}{K^2} \quad (11)$$

which always holds as long as $K > 1$. Thus, in (4) we neglect the positive and bounded term.

III. SUPPLEMENTARY FIGURES

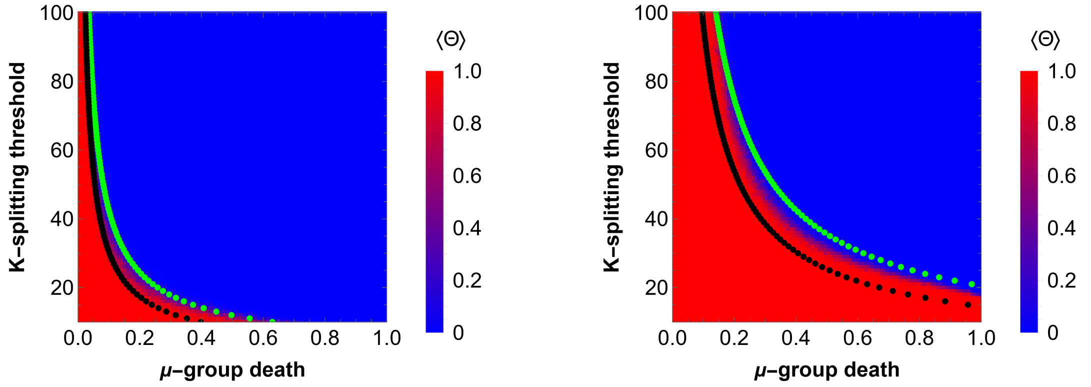

(a) (b)

Supplementary Figure 1: Survival of groups for different values of model parameters. The counterpart of Fig.1A for $N_g = 25$ and $N_g = 100$, a) and b), respectively. All the remaining parameters are the same as in the Fig.1A.

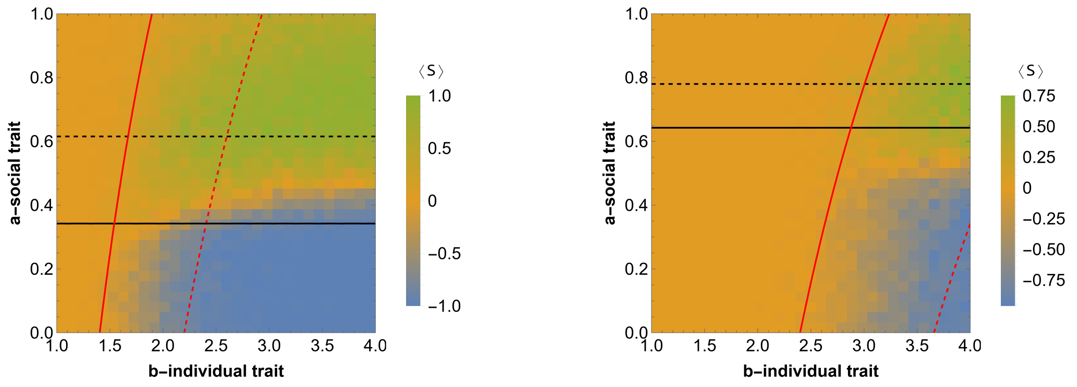

(a) (b)

Supplementary Figure 2: Competition outcome between groups of social and asocial individuals in the case of relative fitness advantage. The counterpart of Fig.2A for $N_{g,A} = \frac{N_g}{4}$, and $K = 10$ and $K = 15$, respectively. The remaining parameters are the same as in the Fig.2A.

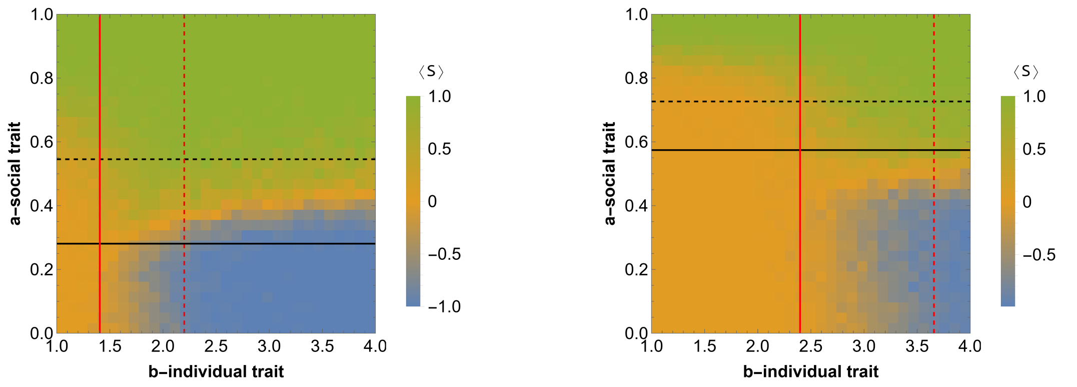

(a) (b)

Supplementary Figure 3: Competition outcome between groups of social and asocial individuals in the case of absolute fitness advantage. The counterpart of Fig.4A for $N_{g,A} = \frac{N_g}{4}$, and $K = 10$ and $K = 15$, respectively. The remaining parameters are the same as in the Fig.4A.
